# Convalescence after Appendicitis

**Published:** 1902-07-26

**Authors:** 


### CONVALESCENCE AFTER APPENDICITIS.

In the course of a clinical lecture on Appendicitis, delivered at St. George's Hospital, Sir William Bennett[1] discussed the question What period must elapse, after an operation in an ordinary case treated in the quiescent stage, before the patient may get about and begin to resume his ordinary occupation? This is always a difficult question to answer exactly, and it is necessary to make one's estimate sufficiently wide to leave a good margin. Speaking generally, a man who has had an operation for appendicitis, no matter how well he feels, or how well he appears, or how tight the scar seems, should not take any exercise for at least three months. This may seem to him a long time, but, says Sir William, "I have now had a large experience of these cases, and I am sure, if you wish to do the very best for your patient, you should not let him do anything beyond gentle exercise for at least six months." At the end of that time a labourer, for example, may resume his work, but certainly unless he is driven to it by great emergency there should be no attempt to resume heavy manual labour at any earlier date. "Indeed, it is quite certain that a man cannot consider himself *absolutely safe for heavy work* after an operation for appendicitis for twelve months." Thus it may be laid down that in three months a man may begin to resume his ordinary occupation in a mild way. At the end of six months he may resume ordinary work which is not very heavy, but he must be prepared to slack off if he finds any pain or irritation about the part, and at the end of a year he may consider himself sound for any purpose. The dangers connected with too early exercise seem to depend in part upon the scar which, until it is hard, will not bear any great tension, partly on the adhesions which often form around the stump of the appendix, and require very gradual stretching, any violent exertion in the early stages being likely to tear them and lead to very serious mischief, and lastly upon the atonic conditions of the cæcum which is apt to persist for a time after an attack of appendicitis.

[1] Clinical Journal, July 9.

